# Adaptive and variable intraguild predators facilitate local coexistence in an intraguild predation module

**DOI:** 10.1186/1472-6785-12-6

**Published:** 2012-05-24

**Authors:** San-He Wu, Toshinori Okuyama

**Affiliations:** 1 Department of Entomology, National Taiwan University, No. 1, Sec. 4, Roosevelt Rd, Taipei, 106, Taiwan

**Keywords:** Invasion analysis, Stability analysis, Behavioral variation, Coexistence, Omnivory

## Abstract

**Background:**

Intraguild predation (IGP) is common in nature, but its ecological role is still illusive. A number of studies have investigated a three species IGP module that consists of an intraguild predator, intraguild prey, and resource species in which the intraguild predator and the intraguild prey exploitatively compete for the resource while the intraguild predator also consumes the intraguild prey. A common prediction of models of the IGP module is that the coexistence of the species is difficult, which is considered inconsistent to the ubiquity of IGP in nature. This study revisits the IGP module and provides an alternative coexistence mechanism by focusing on a commonly used analysis method (i.e., invasion analysis) in light of individual variation in adaptive behavior.

**Results:**

Invasion analysis underestimates the possibility of coexistence regardless of the presence or absence of adaptive behavior. Coexistence is possible even when invasion analysis predicts otherwise. The underestimation by invasion analysis is pronounced when the intraguild predator forages adaptively, which is even further pronounced when the expression of foraging behavior is variable among intraguild predators.

**Conclusions:**

The possibility of coexistence in the IGP module is greater than previously thought, which may have been partly due to how models were analyzed. Inconsistent conclusions may result from the same model depending on how the model is analyzed. Individual variation in adaptive behavior can be an important factor promoting the coexistence of species in IGP modules.

## Background

Intraguild predation (IGP) is where predation occurs among predators of the same guild and is one of the most frequently observed species interactions [1,2]. Predators are considered to belong to the same guild when they consume similar resources [3]. The role of IGP in general ecological dynamics as well as in applied fields such as agriculture and conservation has been studied [4-6]. A commonly studied IGP module consists of three species: intraguild predator (IGpredator hereafter), intraguild prey (IGprey hereafter), and resource species. In the module, the IGpredator and IGprey exploitatively compete for the resource while the IGpredator also consumes the IGprey. Mathematical models of the IGP module predict that coexistence between the IGpredator and IGprey is difficult e.g. [7] despite the ubiquity of IGP in nature. This apparent discrepancy resulted in a number of theoretical studies focusing on coexistence mechanisms [7-13], which also is the focus of this study.

Organisms exhibit a variety of behavioral expressions that are thought to enhance their fitness. The importance of such adaptive behaviors has been recognized in a variety of ecological food web modules including ones with IGP [8,13-16]. For example, in the IGP module, an optimal prey choice behavior of the IGpredator can enhance the coexistence [8]. Similarly, antipredator behavior of the IGprey also enhances the coexistence [13]. However, in these studies, the positive effects of the adaptive behavioral expressions on coexistence are small. One important missing factor in previous studies of adaptive behavior is between-individual variation. An assumption of typical adaptive behavior models is that behavioral variation among individuals is negligible or such variation does not affect community dynamics. However, these assumptions are generally inappropriate, especially in nonlinear ecological processes [17]. Thus, although the roles of adaptive behaviors in IGP modules have been studied, the robustness of the results to individual variation is not known.

Mechanisms of coexistence in food web modules (including ones with IGP) are commonly studied using invasion analysis (also known as invasibility analysis) e.g. [10,18]. In an invasion analysis of the IGP module, mutual invasibility is interpreted as coexistence. (Invasion analysis and mutual invasibility are discussed in detail below.) However, such mutual invasibility is a condition for persistence. Even if a system is not persistent (i.e., if there exist an initial population densities such that the corresponding trajectory leads to extinction of one or more species), there still can be locally stable attractors [19-22]. In other words, if initially population densities are in the domain of attraction for a local attractor, the species will coexist along this attractor. Therefore, existing results of invasion analyses do not necessarily indicate that coexistence is strictly impossible.

To investigate the coexistence of the species in the IGP module, this study considers two factors: analysis methods and individual variation in adaptive behavior. We show that coexistence is possible even when mutual invasibility does not hold. Subsequently, we show that when adaptive behavior and individual variation are considered, invasion analysis further underestimates the likelihood of coexistence; individual variation in adaptive behavior can substantially enhance the coexistence of the IGpredator and IGprey.

## Methods

### Models

We follow Křivan and Diehl [8] who considered the IGP module in which the dynamics of the IGpredator density *P*, IGprey density *N*, and resource density *R*, are described by

(1) $$\frac{\text{d}R}{\text{d}t}=R\left(r\left(1-\frac{R}{K}\right)-\frac{\lambda_{\mathrm{RN}}N}{1+h_{\mathrm{RN}}\lambda_{\mathrm{RN}}R}-\frac{u_{\mathrm{RP}}\lambda_{\mathrm{RP}}P}{1+u_{\mathrm{RP}}\lambda_{\mathrm{RP}}h_{\mathrm{RP}}R+u_{\mathrm{NP}}\lambda_{\mathrm{NP}}h_{\mathrm{NP}}N}\right)$$

(2) $$\frac{\text{d}N}{\text{d}t}=N\left(\frac{e_{\mathrm{RN}}\lambda_{\mathrm{RN}}R}{1+h_{\mathrm{RN}}\lambda_{\mathrm{RN}}R}-\frac{u_{\mathrm{NP}}\lambda_{\mathrm{NP}}P}{1+u_{\mathrm{RP}}\lambda_{\mathrm{RP}}h_{\mathrm{RP}}R+u_{\mathrm{NP}}\lambda_{\mathrm{NP}}h_{\mathrm{NP}}N}-m_{N}\right)$$

(3) $$\frac{\text{d}P}{\text{d}t}=P\left(\frac{e_{\mathrm{RP}}u_{\mathrm{RP}}\lambda_{\mathrm{RP}}R+e_{\mathrm{NP}}u_{\mathrm{NP}}\lambda_{\mathrm{NP}}N}{1+u_{\mathrm{RP}}\lambda_{\mathrm{RP}}h_{\mathrm{RP}}R+u_{\mathrm{NP}}\lambda_{\mathrm{NP}}h_{\mathrm{NP}}N}-m_{P}\right)$$

where *r* is the intrinsic growth rate of the resource, *K* is the carrying capacity, *λ*_*ij*_ is the encounter rate of species *j* for species *ie*_*ij*_ is the efficiency of converting energy of species *i* for species *jh*_*ij*_ is the handling time of species *j* for species *i*, and *m*_*i*_ is the density-independent mortality rate of species *i*. *u*_*iP*_ is the probability that the IGpredator attacks species *i* upon an encounter. The fixed behavior model assumes that *u*_*NP*_ = 1 and *u*_*RP*_ = 1 (i.e., the IGpredator always attacks the IGprey and the resource).

Křivan and Diehl [8] considered that the IGpredator optimally chooses its prey according to a prey choice model [23]. The solution to this problem is well known [24] and is as follows. Suppose the IGprey is more profitable than the resource (*e*_*NP*_/*h*_*NP*_ > *e*_*RP*_/*h*_*RP*_), the IGpredator always attacks the IGprey (i.e., *u*_*NP*_ = 1) when it encounters an IGprey. The IGpredator also always attacks the resource upon an encounter (i.e., *u*_*RP*_ = 1) when the the IGprey density is below a threshold density *N*_*T*_ = *e*_*RP*_/(*λ*_*NP*_*h*_*NP*_*h*_*RP*_(*e*_*NP*_/*h*_*NP*_*e*_*RP*_/*h*_*RP*_)) but always ignores the resource (i.e., *u*_*RP*_ = 0) otherwise. Similarly, when the resource is more profitable than the IGprey (*e*_*RP*_/*h*_*RP*_ > *e*_*NP*_/*h*_*NP*_), the IGpredator always attacks the resource (i.e., *u*_*RP*_ = 1) and also always attacks the IGprey (i.e., *u*_*NP*_ = 1) only when the resource density is below a threshold density *R*_*T*_ = *e*_*NP*_/(*λ*_*RP*_*h*_*RP*_*h*_*NP*_(*e*_*RP*_/*h*_*RP*_*e*_*NP*_/*h*_*NP*_)) and entirely reject the IGprey (i.e., *u*_*NP*_ = 0) when the resource density is above the threshold density (*R* >  *R*_*T*_).

We consider a model which incorporates individual variation in the prey choice behavior where IGpredators have variable perceptions about the densities of the interacting species. In our model, individual IGpredators do not show partial preference [12,25,26]. Suppose only a fraction of IGpredators attack the resource at a given condition. In our model, this occurs because some IGpredators always attack the resource while the rest always ignore the resource (i.e., individual variation). On the other hand, in partial preference models, this occurs because all IGpredators attack the resource with the probability equals to the observed fraction of IGpredators that are attacking the resource (i.e., no individual variation).

The optimal behavioral expression (the all-or-nothing behavior determined by *u*_*RP*_ and *u*_*NP*_) depends on the density of the more profitable prey of the two, and thus perceptual variation in the density leads to variable behavioral expressions among IGpredators [27]. Because perceived densities take non-negative continuous values, we use a gamma distribution gamma(*αβ*) to describe their distribution. By specifying the mean *μ* and variance *σ*^2^ of the distribution, *α* and *β* can be described as *α* =  *μ*^2^/*σ*^2^ and *β* =  *σ*^2^/*μ*. We assume that the mean is the true density (e.g. *μ* =  *N* if *e*_*NP*_/*h*_*NP*_ > *e*_*RP*_/*h*_*RP*_). We also assume that the perceptional variance is the same as the mean (*σ*^2^ = *μ*). Then the dynamics of the IGP module with individual variation can be described by,

(4) $$\frac{\text{d}R}{\text{d}t}=R\left(r\left(1-\frac{R}{K}\right)-\frac{\lambda_{\mathrm{RN}}N}{1+h_{\mathrm{RN}}\lambda_{\mathrm{RN}}R}-\frac{\lambda_{\mathrm{RP}}Pq_{\mathrm{RN}}}{1+\lambda_{\mathrm{RP}}h_{\mathrm{RP}}R+\lambda_{\mathrm{NP}}h_{\mathrm{NP}}N}-\frac{\lambda_{\mathrm{RP}}Pq_{R}}{1+\lambda_{\mathrm{RP}}h_{\mathrm{RP}}R}\right)$$

(5) $$\frac{\text{d}N}{\text{d}t}=N\left(\frac{e_{{}_{\mathrm{RN}}}\lambda_{\mathrm{RN}}R}{1+h_{\mathrm{RN}}\lambda_{\mathrm{RN}}R}-\frac{\lambda_{\mathrm{NP}}Pq_{\mathrm{RN}}}{1+\lambda_{\mathrm{RP}}h_{\mathrm{RP}}R+\lambda_{\mathrm{NP}}h_{\mathrm{NP}}N}-\frac{\lambda_{\mathrm{NP}}Pq_{N}}{1+\lambda_{\mathrm{NP}}h_{\mathrm{NP}}N}-m_{N}\right)$$

(6) $$\frac{\text{d}P}{\text{d}t}=P\left(q_{R}\frac{e_{\mathrm{RP}}\lambda_{\mathrm{RP}}R}{1+\lambda_{\mathrm{RP}}h_{\mathrm{RP}}R}+q_{N}\frac{e_{\mathrm{NP}}\lambda_{\mathrm{NP}}N}{1+\lambda_{\mathrm{NP}}h_{\mathrm{NP}}N}+q_{\mathrm{RN}}\frac{e_{\mathrm{RP}}\lambda_{\mathrm{RP}}R+e_{\mathrm{NP}}\lambda_{\mathrm{NP}}N}{1+\lambda_{\mathrm{RP}}h_{\mathrm{RP}}R+\lambda_{\mathrm{NP}}h_{\mathrm{NP}}N}-m_{P}\right)$$

where *q*_*RN*_*q*_*R*,_*q*_*N*_ are the fraction of IGpredators that attacks both the resource and the IGprey, the resource only, and the IGprey only, respectively. For example, when the IGprey is more profitable than the resource, *q*_*R*_ = 0. *q*_*RN*_ depends on the perceived density *x* of the profitable prey *N* and is,

(7) $$q_{\mathrm{RN}}=\int_{0}^{N_{T}}f\left(x\right)dx$$

where *f*( *x*) is the gamma distribution discussed above. In other words, *q*_*RN*_ is the proportion of IGpredators that perceives the density of the IGprey is less than the threshold density *N*_*T*_. Because individual IGpredators either perceive that the density of the IGprey is greater than the threshold or not (i.e., one or the other), the proportion of IGpredators that perceive that the density of the IGprey is above the threshold (*q*_*N*_) is 1 − *q*_*RN*_. Similarly, when the resource is more profitable (which leads to *q*_*N*_ = 0), and the proportion of IGpredators that perceives that the density of the resource is less than the threshold density, *R*_*T*_ is,

(8) $$q_{\mathrm{RN}}=\int_{0}^{R_{T}}f\left(x\right)dx$$

followed by *q*_*R*_ = 1 − *q*_*RN*_*.*

The effect of adaptive behavior and individual variation on coexistence is examined using invasion and stability analyses supplemented with numerical simulations. The parameter values used in the analyses follows a previous study for comparison [8]: *r* = 0.3, *λ*_*RN*_ = 0.037, *λ*_*RP*_ = 0.025, *λ*_*NP*_ = 0.025, *h*_*RN*_ = 3, *h*_*RP*_ = 4, *h*_*NP*_ = 4, *e*_*RN*_ = 0.6, *e*_*RP*_ = 0.36, *e*_*NP*_ = 0.6, *m*_*N*_ = 0.03, *m*_*P*_ = 0.0275.

### Invasion analysis

In this invasion analysis, we examine whether the IGpredator can invade communities that consist of the IGprey and resource, and whether the IGprey can invade communities that consist of the IGpredator and resource. When both the IGpredator and IGprey can invade each other (i.e., mutually invasible), coexistence is implied.

Suppose when resident communities are at equilibrium, the invasion conditions for the IGprey and IGpredator, respectively, are,

(9) $$\frac{\text{d}N}{\text{d}t}{\frac{1}{N}|}_{N=0}=\frac{e_{\mathrm{RN}}\lambda_{\mathrm{RN}}R_{\mathrm{RP}}^{*}}{1+h_{\mathrm{RN}}\lambda_{\mathrm{RN}}R_{\mathrm{RP}}^{*}}-\frac{u_{\mathrm{NP}}\lambda_{\mathrm{NP}}P_{\mathrm{RP}}^{*}}{1+u_{\mathrm{RP}}\lambda_{\mathrm{RP}}h_{\mathrm{RP}}R_{\mathrm{RP}}^{*}}-m_{N}$$

(10) $$\frac{\text{d}P}{\text{d}t}{\frac{1}{P}|}_{P=0}=\frac{u_{\mathrm{RP}}\lambda_{\mathrm{RP}}e_{\mathrm{RP}}R_{\mathrm{RN}}^{*}+u_{\mathrm{NP}}\lambda_{\mathrm{NP}}e_{\mathrm{NP}}N_{\mathrm{RN}}^{*}}{1+u_{\mathrm{RP}}\lambda_{\mathrm{RP}}h_{\mathrm{RP}}R_{\mathrm{RN}}^{*}+u_{\mathrm{NP}}\lambda_{\mathrm{NP}}e_{\mathrm{NP}}N_{\mathrm{RN}}^{*}}-m_{P}$$

where $R_{\mathrm{RP}}^{*}$ and $P_{\mathrm{RP}}^{*}$ are the equilibrium densities of the resource and IGpredator in the resouce-IGpredator community; $R_{\mathrm{RN}}^{*}$ and $N_{\mathrm{RN}}^{*}$ are the equilibrium densities of the resource and the IGprey in the resource-IGprey community, respectively. When the expressions in Equations (9) and (10) are both positive, mutually invasibility is established. When resident communities exhibit cycles, the invasibility conditions of the IGprey and IGpredator, respectively, are,

(11) $$\frac{1}{\tau_{\mathrm{RP}}}{\int_{0}^{\tau_{\mathrm{RP}}}\frac{\text{d}N}{dt}\frac{1}{N}|}_{N=0}dt$$

(12) $$\frac{1}{\tau_{\mathrm{RN}}}{\int_{0}^{\tau_{\mathrm{RN}}}\frac{\text{d}P}{dt}\frac{1}{P}|}_{P=0}dt$$

where τ_*RP*_ and τ_*RN*_ are the periodicity of the cycle for the resource-IGpredator and the resource-IGprey resident communities, assuming resident populations are on the trajectory of the limit cycles.

The inclusion of individual variation does not affect results of the invasion analysis. The possibility of invasion of the IGprey is not affected by individual variation because the perceptual variance is the same as the true density (e.g., at an invasion event, the variance is 0). The possibility of invasion of the IGpredator is also unaffected by individual variation if we assume that some invading individuals exhibit optimal behavior.

### Stability analysis

In invasion analysis, when the mutual invasibility condition is not met, coexistence is considered impossible [28]. However, this is not always true [19-22]. To examine the possibility of coexistence that cannot be studied with invasion analysis, equilibrium stability is also examined using the standard Routh-Hurtwitz condition of the Jacobian matrix evaluated at equilibria [29]. When a coexistence equilibrium (stable or unstable) does not exist, coexistence is considered impossible. When an equilibrium is unstable, the possibility of coexistence through limit cycles is examined numerically. This is done by simulating the model at a point near the equilibrium (1% equilibrium perturbation in the three densities). Although the local stability of the fixed behavior model and the adaptive behavior model without individual variation can be analyzed with their explicit equilibrium solutions, explicit equilibrium expressions could not derived when individual variation was considered. For the analysis of the model with individual variation, stability and limit cycle possibilities were analyzed with numerical simulations.

## Results

### Invasion analysis

**Figure 1:**
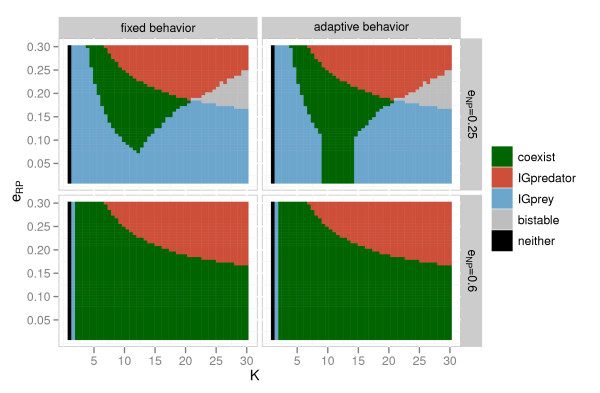
**Effect of the resource profitability to the IGpredator (*e***_***RP***_**) and carrying capacity (*K*) on the dynamics of IGP module based on invasion analysis.** Mutual invasibility is supported in the region coded ‘coexist’. In the region of ‘IGpredator’, IGpredators are expected to win. In the region of ‘IGprey’, IGprey are expected to win. In the ‘bistable’ region, outcomes of the species interaction depend on initial densities. When the carrying capacity is very low, ‘neither’ species can be sustained.

The results of invasion analysis are the same as those shown in Křivan and Diehl [8] except that the carrying capacity *K* is further extended in the current analysis (Figure 1). When the IGprey is highly beneficial to the IGpredator (*e*_*NP*_ = 0.6), there is little difference between the fixed behavior model and the adaptive behavior model. When the IGprey is less profitable (*e*_*NP*_ = 0.25), the possibility of coexistence is extended in the adaptive behavior model, though by a small margin [8]. Coexistence is predicted entirely impossible at high productivities when *e*_*NP*_ = 0.25.

### Stability analysis

**Figure 2:**
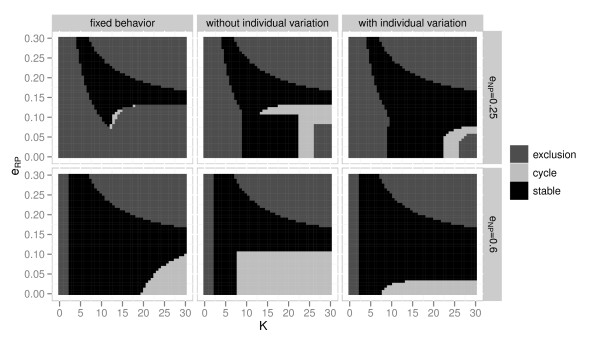
**Effect of the resource profitability to the IGpredator (*e***_***RP***_**) and carrying capacity (*K*) on the dynamics of IGP module based on stability analysis.** There are three outcomes: coexistence is not possible (exclusion), coexist through cycles (cycle), and stable coexistence (stable).

Even when invasion analysis predicts that the IGpredator will be excluded (Figure 1), stability analysis shows that they can coexist stably and/or through limit cycles in large parameter regions (Figure 2). That is, invasion analysis underestimates the possibility of coexistence. This difference between the analysis methods is most notable when *e*_*NP*_ = 0.25.

The inclusion of adaptive behavior enhances coexistence both in invasion and stability analyses, but adaptive behavior shows a stronger positive effect in the latter analysis. In other words, the underestimated coexistence regions are larger in the models with adaptive behavior. When *e*_*NP*_ = 0.25, invasion analysis predicts that coexistence is impossible at high productivities (Figure 1), but stability analysis predicts it is possible at high values of *K* (e.g. *K* = 100, although results are shown only up to *K* = 30 in Figure 2) in all models. In comparison between the adaptive behavior models with and without individual variation, the model with individual variation tends to stabilize non-equilibrium dynamics.

**Figure 3:**
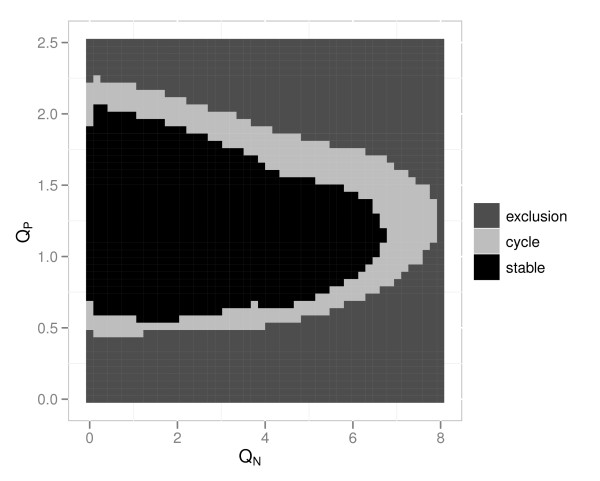
**A domain of attraction in the adaptive behavior model without individual variation.** The initial densities of the resource, IGprey and IGpredator are ( R^*^, N^*^Q_*N*_, P^*^Q_*P*_) where R^*^, N^*^, P^*^ are the equilibrium densities of the resource, IGprey and IGpredator, respectively. *Q*_*N*_ and *Q*_*P*_ represent perturbation to the equilibrium densities of the IGprey and IGpredator such that *Q*_*N*_ = *Q*_*P*_ = 1 represents no perturbation. There are three outcomes. The community returns to the equilibrium (stable). The community does not return to the equilibrium but exhibit cycles and persist (cycle). The community does not persist (i.e., an exclusion will occur). *K* = 30, *e*_*RP*_ = 0.15, *e*_*NP*_ = 0.25.

**Figure 4:**
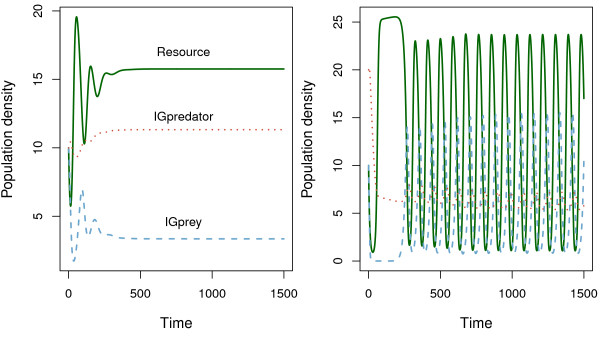
**Community dynamics of the adaptive behavior model without individual variation under different initial densities: (*R*,*N*,*P*) = (10,10,10) [left figure] and (10,10,20) [right figure].***K* = 30, *e*_*RP*_ = 0.15, *e*_*NP*_ = 0.25.

If internal attractors (e.g. stable equilibrium) have small domains of attraction, the difference between the analysis methods may not be ecologically significant because the community dynamics can easily move away from the attractors due to inherent ecological stochasticity. In our model, internal attractors usually have wide domains of attraction (Figure 3). When the initial densities of the IGprey and IGpredator are systematically changed from their equilibrium densities, communities would return to the equilibrium if the changes are small. When a community is strongly perturbed from the equilibrium, it may persist through cycles. When a community is even more strongly perturbed, a species may be excluded. This result shows the presence of multiple attractors. For example, when stable coexistence is indicated in Figure 2, both stable coexistence and cyclic coexistence may be possible (Figure 4).

## Discussion

Although IGP is common in nature, models often predict that it is unlikely. This study shows that this discrepancy can be partly due to how models are analyzed. Invasion analysis, though commonly used, may predict that coexistence is impossible when it may be readily possible through locally stable attractors (e.g. stable equilibrium). In fact, locally stable attractors exist in wide parameter regions when individual variation in adaptive behavior is considered. These results suggest that both individual variation and analysis methods are important for examining the dynamics of the IGP module.

The result about the analysis methods (i.e. mutual invasibility is not necessary for coexistence) is valid no matter which model is considered. In other words, neither adaptive behavior nor individual variation is necessary. For example, the species can coexist even at very high productivity levels without an additional ecological factor (i.e., fixed behavior without individual variation). However, the coexistence possibility further expands with inclusions of adaptive behavior and individual variation. This difference between invasibility and coexistence exists due to nonlinear interactions (i.e., saturating functional responses). The nonlinear functions allow the IGP module to have alternative equilibria (e.g. all three species present and only two species present) [1,7,30]. When all per capita interactions are linear functions, internal attractors would not exist when the mutual invasibility fails.

One may argue that invasion analysis is a more conservative way to study coexistence. For example, even if an internal attractor exists (i.e., coexistence is possible), a community may not persist if it experiences a strong perturbation. However, the domain of attraction is not negligible (Figure 3). When considering why ecological communities in the field can persist even though models predict otherwise, the existence of internal attractors cannot be ignored. In addition to being conservative, another reason one might focus on invasibility is that communities are assembled by invasions, and initially colonizing species are likely rare. In invasion analysis, this is modeled by assuming the density of an invading species is 0 (i.e., completely negligible). In ecological invasions with finite population sizes, however, only few invading individuals can substantially violate the assumption. Results of invasion analysis can change if this assumption is slightly relaxed (i.e., the density of an invading species is assumed to be very small but not nil). Under these scenarios, internal attractors become more relevant to understanding real ecological dynamics.

The interpretation of the effect of adaptive foraging in the IGP module is not straightforward. Here we make two comparisons. One comparison is between the fixed behavior model and the adaptive behavior model without individual variation. This comparison shows that the inclusion of optimal foraging enhances the possibility of coexistence (Figures 2 and 3). The other comparison is between the adaptive behavior models with and without individual variation, which shows that individual variation enhances coexistence and also stabilizes non-equilibrium dynamics. Because individual variation reduces the average per-capita fitness of the IGpredator, these two comparisons show qualitatively different results. The former implies that optimal foraging has a positive effect, and the latter comparison result implies that optimal foraging has a negative effect on coexistence. When both fixed behavior and perfect adaptive behavior do not allow the community to persist, individual variation may be able to give the balance needed for the community to persist. Similar results relating to suboptimal behavior enhancing coexistence exist in other food web modules [27,31]. Optimal foraging behavior of individuals leading to extinction of the population has also been reported in other studies e.g. [32].

Abrams and Fung [12] studied the IGP module in which the IGpredator exhibits partial preference while this study focused on individual variation (difference between partial preference and individual variation is discussed above), and both studies show that sub-optimal adaptive behavior can increase coexistence possibilities. Because of some differences between these studies Abrams and Fung (e.g. [12] mainly focused on invasion criteria while this study focused on internal attractors, and the two studies consider different parameter values), it is difficult to directly compare differences between partial preference vs. individual variation. However, it has been reported in a study on a different food web module that the difference between partial preference and individual variation can cause ecologically significant differences [27]. Although (empirical) studies have commonly considered individual variation as a factor to account for the type I error in statistical hypothesis tests, characterizing the patterns of variation will likely to provide rich information to studying ecological dynamics.

## Conclusions

Individual variation in adaptive behavior can substantially enhance coexistence in the IGP module, and model predictions can be quite different depending on how they are analyzed. The findings reduce the mismatch between the ubiquity of IGP modules in nature and modeled coexistence. Although the inadequacy of invasion analysis has been known for a long time, we do not know how common it may be (e.g. in other food web modules) and suspect that such deviations may be more common especially when adaptive behavior and/or individual variation are considered.

## Competing interests

The authors declare that they have no competing interests.

## Authors’ contributions

WSH and TO analyzed the models and wrote the manuscript. Both authors read and approved the final manuscript.
